# Land-Use/Land-Cover Change from Socio-Economic Drivers and Their Impact on Biodiversity in Nan Province, Thailand

**DOI:** 10.3390/su11030649

**Published:** 2019-01-26

**Authors:** Yongyut Trisurat, Hiroaki Shirakawa, John M. Johnston

**Affiliations:** 1 Faculty of Forestry, Kasetsart University, Bangkok 10900, Thailand; 2 Department of Urban Environment, Graduate School of Environmental Studies, Nagoya University, Nagoya 464-8601, Japan;; 3 USEPA/ORD/NERL Computational Exposure Division, Athens, GA 30605, USA;

**Keywords:** biodiversity, forest cover target, land-use change, land-use scenarios, socio-economic-drivers, vulnerable areas

## Abstract

The rate of deforestation declined steadily in Thailand since the year 2000 due to economic transformation away from forestry. However, these changes did not occur in Nan Province located in northern Thailand. Deforestation is expected to continue due to high demand for forest products and increased agribusiness. The objectives of this paper are (1) to predict land-use change in the province based on trends, market-based and conservation scenarios, (2) to quantify biodiversity, and (3) to identify biodiversity hotspots at greatest risk for future deforestation. This study used a dynamic land-use change model (Dyna-CLUE) to allocate aggregated land demand for three scenarios and employed FRAGSTATS to determine the spatial pattern of land-use change. In addition, the InVEST Global Biodiversity Assessment Model framework was used to estimate biodiversity expressed as the remaining mean species abundance (MSA) relative to their abundance in the pristine reference condition. Risk of deforestation and the MSA values were combined to determine biodiversity hotspots across the landscape at greatest risk. The results revealed that most of the forest cover in 2030 would remain in the west and east of the province, which are rugged and not easily accessible, as well as in protected areas. MSA values are predicted to decrease from 0.41 in 2009 to 0.29, 0.35, and 0.40, respectively, under the trends, market-based and conservation scenarios in 2030. In addition, the low, medium, and high biodiversity zones cover 46, 49 and 6% of Nan Province. Protected areas substantially contribute to maintaining forest cover and greater biodiversity. Important measures to protect remaining cover and maintain biodiversity include patrolling at-risk deforestation areas, reduction of road expansion in pristine forest areas, and promotion of incentive schemes for farmers to rehabilitate degraded ecosystems.

## 1. Introduction

Thailand is geographically located in the core of the Greater Mekong Sub-region (Cambodia, Lao People’s Democratic Republic (PDR), Myanmar, Thailand, Vietnam, and China-Yunnan), with favorable prevailing climate and productive soils. Thailand has long been recognized as one of the world’s top rice exporters [1] and plays an important role in maintaining food supplies and providing food security around the world [2]. The country’s economy evolved in four stages and was largely an outgrowth of the agricultural sector. The first stage was regarded as the subsistence farming economy, where people grew commodity crops for domestic consumption and limited international trading [3]. The second stage during the 1^st^–4^th^ National Economic and Social Development Plan or NESDP (1961–1981) [4] focused on commercial crops for international trade. This national policy resulted in a rapid increase of cultivated land area by two-fold, from 15% in 1961 to 31% in 1980 [5]. In addition, the agricultural sector contributed more than 25% of the Gross Domestic Product (GDP) of Thailand [6] and more than 70% of the total population worked in the agricultural sector [7].

Since 1980, Thailand’s national policies have shifted from an agriculture-based economy to manufacturing and service sectors. Thailand is currently in the fourth stage (Thailand 4.0) in which the economy moved towards value-based and innovative products [8]. The contribution of the service sector now accounts for over 50% of the total GDP. In contrast, the contribution of the agricultural sector dropped below 10% after 1990 [6]. Nevertheless, a large proportion of the labor force (56% of total population) is involved in the agricultural sector [7].

Agricultural and economic development policies resulted in both positive and negative consequences. In addition, agricultural policies largely maximize economic return, while degrade biodiversity and ecosystem services in most developing countries [9,10]. A substantial increase in agricultural income is mainly due to cultivation of forest land. Forest cover declined from 53.3% of the country in 1961 to 25.1% in 1999 [11], despite the nationwide logging ban imposed in 1989 [12]. A recent assessment spanning 2000–2016 revealed that forest cover in Thailand stabilized at 31–33%, largely due to economic transformation and strict law enforcement [11]. However, forest land was not converted at the same rate and evenly distributed across the country. The decreased deforestation rate (8.1%) was observed in northern Thailand during 2000–2016, and the greatest rate of 16% was reported in Nan province followed by Chiang Mai province (12%) in northern Thailand. In contrast, the remaining forest cover in central and southern Thailand stabilized during this period [11].

Deforestation caused by the expansion of agricultural land and accompanying infrastructure development increased pressure on biodiversity and ecosystem services. In addition to diminishing suitable habitat for flora and fauna, deforestation also creates habitat fragmentation, reduces patch size, and isolates suitable habitats [13,14]. Larger mammals are more vulnerable than medium-and small-sized species [15–18]. This is the case for plant diversity as well [19]. Road building provides access for poaching [20] and land encroachment [21], especially near the forest edge. Bird and mammal populations were substantially depleted within 7 and 40 km from access points, and hunting pressure was greater in areas nearby bush meat markets [20]. Furthermore, the consequences of land-use change can also include degradation of watershed services such as the provisioning of clean water and sediment retention [22,23]. Deforestation and its consequences has been listed as an important environmental issue in Thailand [8], Asia and the Pacific region [10], and other geographical regions [24].

Previous and recent measures to protect remaining forest cover (e.g., setting forest targets, establishment of protected areas, reforestation, agricultural land-use planning) are ad-hoc and short-term in most countries in Asia-Pacific region [10,12]. For instance, forest cover targets have been set, but many countries are failing to meet these quantitative goals defined by policy makers [10]. In addition, the Aichi target 11 was defined to encourage the parties to the Convention on Biological Diversity to conserve at least 17% of the terrestrial ecosystems by 2020 [25]. Recently, a half earth for natural reserves was proposed [26] and a few studies were conducted to support [27] and to argue [28] this theory.

Various models have been developed to predict land-use change [29–31] and species distribution [32], ranging from simple approaches to sophisticated simulation systems that incorporate interactions among many model state variables. Generally, land-use change is the result of environmental conditions (e.g., prevailing climate, soil characteristics, and accessibility and suitability for human development) and human drivers (e.g., institutional capacity, technology, human demography, agricultural policies, and land-use restrictions). These drivers are often characterized as multiple interacting factors [31].

Therefore, two research questions motivated this research, including (1) how relevant direct and indirect drivers affect future land use? and (2) to what extent future land use affects biodiversity and guides protection measures? The specific objectives of this study are to predict land-use/land-cover in Nan Province in 2030 using on the synergies between socio-economic and policy drivers, to quantify the consequences of individual and synergies between land-use change and infrastructure development on biodiversity and to determine priority areas for biodiversity protection. Nan Province was selected because it has experienced substantial land-use change in the last decade [11,33], especially the rapid expansion of rubber plantations and maize fields [34]. This was due to the national policy promoting rubber production [35] and the incentives provided to farmers by large companies [35]. In addition, it represents other provinces situated in mountainous areas both in Thailand and other countries, where agriculture remain an economic sector for provincial development [36]. Furthermore, Thai’s government has selected Nan Province as a pilot site to cope with deforestation issues and to promote green growth policy [8].

## 2. Study Area

**Figure 1.**
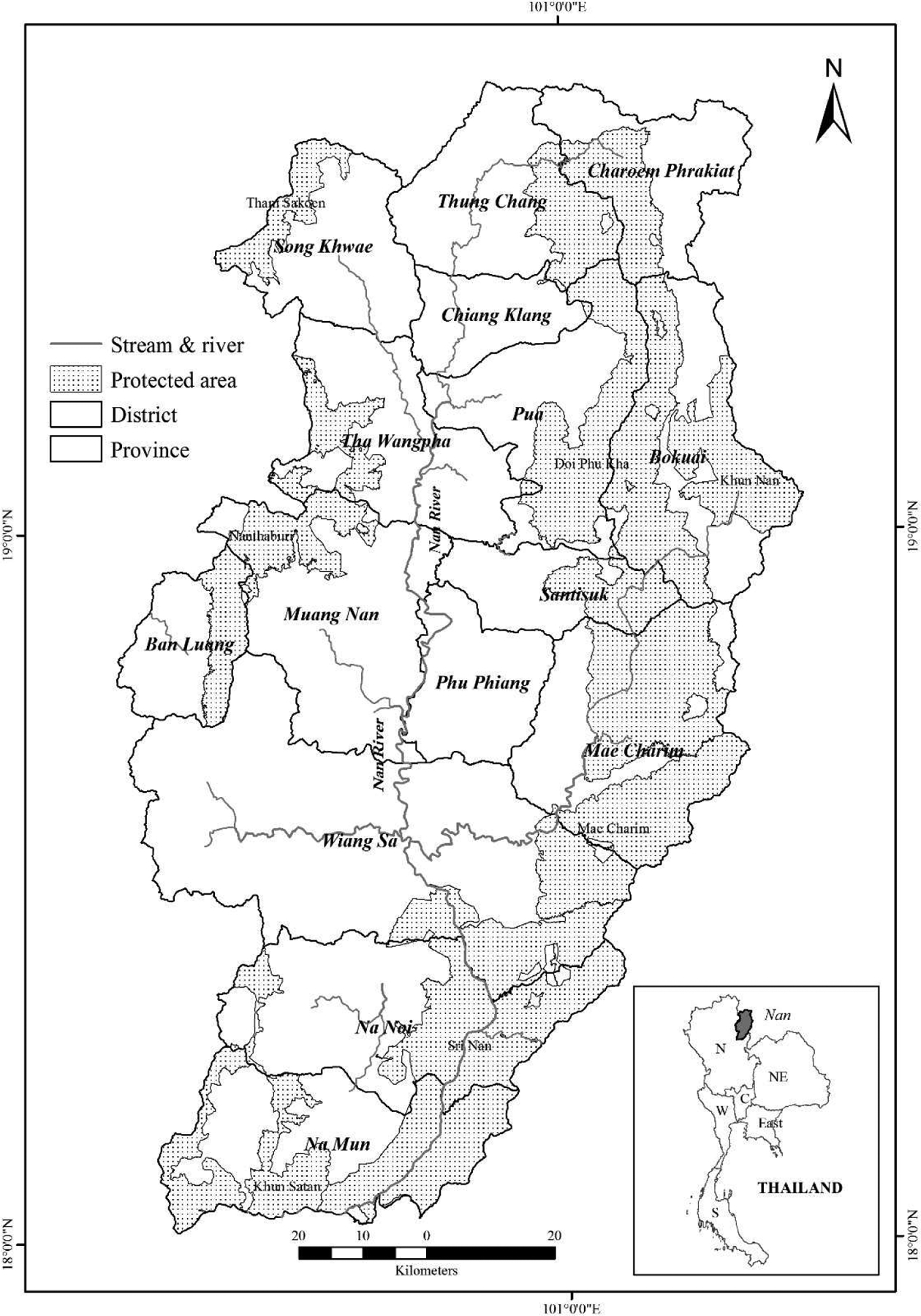
Location of Nan Province and protected areas, Thailand.

Nan is one of the 17 provinces situated in northern Thailand, covering approximately 12,000 km^2^. The province has an elongated shape surrounded by high mountains, except for the southern part (Figure 1). Nan Province accounts for one third of the Nan River Basin, which flows from north to south and joins the Ping, Wang, and Yom Rivers to form the Chao Phraya River in Nakhon Sawan Province. The lowlands (< 400 m) occupy about 18% of the province’s area, while elevations greater than 800 m cover approximately 40%. The highest altitude of 1,980 m above mean sea level is in Doi Phu Kha National Park. The mean annual rainfall during 1961–2013 was 1,268.8 mm. The rainy season begins in May and ends in September. Mean annual temperature is 25.9°C; maximum temperature is 33.1°C, and minimum temperature is 20°C [36].

The population of Nan Province is approximately 478,000 persons, and 55% of the households engage in agriculture. Agricultural sector contributes about 32% of the Gross Provincial Product (GPP) [7]. Mean population density is low at 39 persons/km^2^ compared to the national population density (130 persons/km^2^) due to mountainous topography, which is a barrier to development. An important social problem in the province is poverty. In 2015, 28.8% of Nan’s population lived under the poverty line (USD 1,057 per person per year in 2015), considerably greater than the national proportion of 8.6% [37]. An increased density of households living in poverty was found in steeper terrain because of low income from agriculture and lack of job opportunities.

Agriculture plays a key role in the economy and livelihood of most of the population, particularly tribal people (e.g., Lua, Khmong, Mien and Khmu). Most of the lowlands are dominated by ethnic Thai, whereas the highlands and mountainous territories are populated by other ethnic groups. Land suitable for cultivation is limited: agricultural land in 1977 covered 5.3% of the province. In 2007 agricultural land increased to 10%, with 42% of this area used for maize cultivation. In the last decade, the province faced a maize boom and rapid expansion of rubber plantations. In 2016 maize cultivation covered approximately 127,000 ha, which was nearly fivefold and two-fold greater area than in 2005 and 2007, while the rubber plantations increased over four-fold during 2005–2016 [38,39]. Agricultural land increased to 27.3% of the province’s area, with 13% of this area irrigated.

Long-term monitoring by the Land Development Department (LDD) and the Royal Forest Department (RFD) showed that forest cover in Nan province declined from 92% in 1977 [40] to 61% in 2016 [11]. The greatest deforestation rate of 33% (or 14% per annum) was recorded during 2009–2012 [33]. Protected areas (national parks) and class 1 watersheds cover about 35% and 45% of the area. These two land-use types are recognized as conservation forests [12] and agricultural development, extraction of non-timber products and human settlement are prohibited in these areas. Rapid agricultural expansion driven by favorable market price for maize and government incentives for rubber plantations [35] in the last decade has triggered extensive deforestation in conservation forests.

## 3. Method

The methodology includes four steps (1) defining future land-use scenarios, (2) modeling land-use changes and assessing landscape characteristics, (3) calculating mean species abundance, and (4) assessing biodiversity hotspots at risk of development. Each step is described in more detail below.

### 3.1. Defining Future Land-Use Scenarios

**Figure 2.**
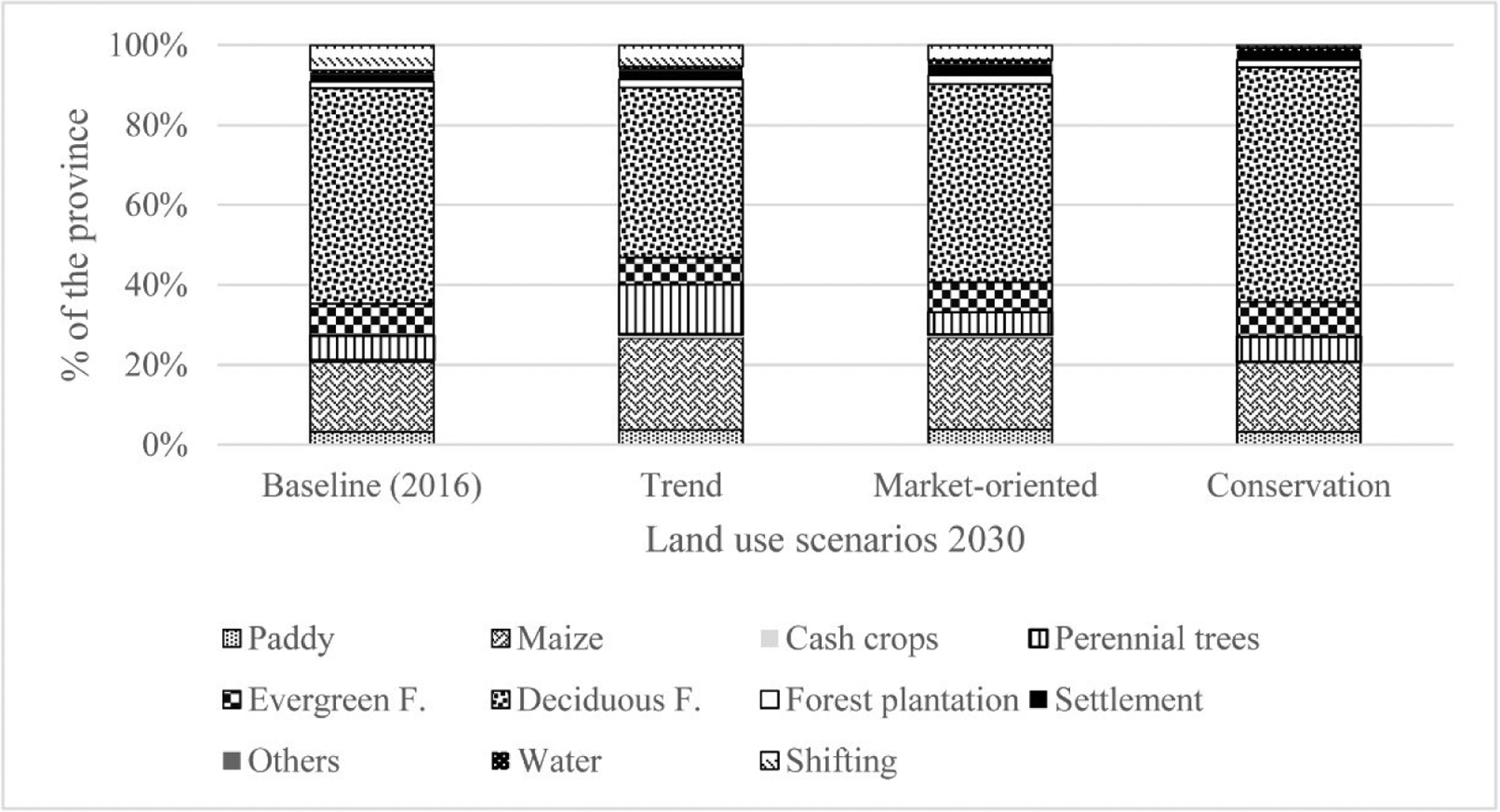
Land-use scenarios in 2030 in Nan Province.

A “scenario” is defined as plausible future in the context of uncertainties, particularly when expected outcomes are highly contingent on indirect and direct effects [41]. There are several methods available to construct scenarios within a decision-making context. These include exploratory, target seeking, ex-ante and ex-post assessment and prior policy screening scenarios. Within the Nan context, a consultation workshop comprising 30 stakeholders from relevant agencies (local and national levels), interested individuals and groups, was conducted in April 2018 to share ideas and information from their collective knowledge. The workshop participants proposed three future land-use scenarios in 2030, called (1) trend, (2) market-oriented, and (3) conservation (Figure 2). These scenarios accommodate and represent various socio-economic drivers, historical and future economic development in Nan Province and ambitious national policies on forest protection and biodiversity conservation. Each scenario prioritizes demand for different land-use types, which are aggregated at the Province level.

The trend scenario was based on a continuation of the land-use change of recent years (2009–2016 [41,42]). The Markov Chain Model [42] predicted that maize area will increase from 17.5% of the province’s area at present to 23.1% in 2030, while natural forests will decrease from 61% in 2016 to 49.3% in 2030. If forest plantation is added, it will reach 51.4% (Figure 2). Perennial trees (mainly rubber plantations) will increase by two-fold from the current level under the assumption of high rubber prices. This scenario also assumed poor forest protection and enforcement, meaning further encroachment inside and outside of protected areas similar to previous trends.

The market-oriented scenario was constructed using target seeking. The Nan Development Plan (2018–2021) aims to achieve a lower rate of land transformation compared to the trend scenario [36]. Furthermore, it targets increased commodity production by 5% per annum from the baseline (2016) to reduce reliance on imported agricultural products from other provinces. With this ambitious target and the assumption of constant agricultural land productivity, the model predicts that forest cover would be less than 30% of the province’s area in 2030 (see equation below).

(1) $${\text{F}}_{\text{n}}={\text{F}}_{0}-\left(\sum_{\text{i}=1}^{\text{j}}{\text{L}}_{\text{i}0}\left[{\left(1+\text{r}\right)}^{\text{n}}-1\right]\right)$$

where F_0_: Forest area at baseline *F*_*n*_: Forest area in year n i: Index of crop *L*_*i*0_: Agricultural area of crop i at baseline r: annual economic growth ratio

Considering constraints on agricultural expansion, the increment rate of 2% per annum was recommended by the workshop participants, resulting in 57% of the province under forest cover in 2030. This scenario assumes 50% of the recent rubber plantations (2014–2016) will be converted to other economic crops, especially maize, and decreased rubber prices. Maize plantation will increase at a rate of 3% annually, half of the recent rate. Increased human settlements (3% annually) and water body class (2% annually) are also expected as the result of tourism trends in the city. Degraded forest will be rehabilitated through agro-forestry and multi-layer cropping practices.

The conservation scenario aims to strictly protect biodiversity in protected areas and maintain watershed services. As mentioned previously, Nan protected areas, cover about 35% of the province’s area, while the watershed class (WSC) 1 accounts for about 46%. The total of these two categories, excluding overlapping areas, is approximately 70% of the province’s area. The Nan Development Plan aims to maintain remaining forest cover (61% of the province’s area) and rehabilitate degraded areas in headwater watersheds at the rate of 2% per annum of the forest area as of 2016 and slightly decline after 2021 [36]. By 2030, total forest land would reach approximately 70%.

### 3.2. Modeling Land-Use Changes and Assessing Landscape Characteristics

The land-use/land-cover (LU/LC) map produced by the Land Development Department in 2016 [42] was used as a baseline for modeling. The original 24 LU/LC classes were generalized to 11 classes, namely (1) paddy fields, (2) maize area, (3) cash crops, (4) perennial trees (fruits and rubber), (5) evergreen forest, (6) deciduous forest, (7) forest plantation, (8) settlement and infrastructure, (9) miscellaneous land uses (others), (10) water body, and (11) shifting cultivation. Paddy is irrigated rice; cash crops include all annual economic crops such as cassava, sugar cane but exclude maize. Perennial trees include fruit trees and rubber plantations, while forest plantation is a mono-tree species plantation such as teak, eucalyptus. Water consists of streams, rivers, lakes, and dams. In addition, shifting cultivation or swidden cultivation is slash and burn forest clearing for planting crops then moving on when soils are depleted (Table 1).

The Dyna-CLUE model (Conversion of Land Use and its Effects Modeling Framework) [45] was selected to predict land-use patterns because the model has been used successfully for allocating future land demands at both local [46,47] and regional levels [48], as well as for dealing with multiple interacting variables [31]. In addition, this model is a spatially explicit and enables to deal with multiple interacting variables [32,35] and restriction policies.

The model requires four inputs: (1) future land-use requirements, (2) characteristics of current land use, (3) spatial policies and restrictions, and (4) land-use type-specific conversion rules. Land-use requirements represent the aggregate land demands of future scenarios (i.e., trends, market-based and conservation). The location preferences of the different LU/LC classes were determined by using logistic regression, identifying the relationship between a particular LU/LC type (dependent variable) and a set of independent variables (drivers).

(2) $$\text{Logit}\left({\text{p}}_{\text{i}}\right)=\text{ln}\left({\text{p}}_{\text{i}}\right)/\left(1-{\text{p}}_{\text{i}}\right)=\beta 0+\beta_{1}{\text{X}}_{1}+\beta_{2}{\text{X}}_{2}+\ldots +\beta_{\text{n}}{\text{X}}_{\text{n}}$$

where p_i_ is the probability of the occurrence of a particular LU/LC type and the X_i_ parameters are the independent variables [45]. βi is the estimated coefficient of each independent variable in the logistic regression. Independent variables that affect land use include soil characteristics, topography (altitude and slope), climate (annual rainfall and mean temperature), and proximity to water (distance to stream), distance from road, total population density (individuals / km^2^), and agricultural population density (households / km^2^). The logistic regression was used because it relates the predicted probabilities of the locations of land use (dependent variable) and a set of location characteristics (independent variables) in which the dependent variable is dichotomous or binary (e.g., paddy or not paddy).

Spatial policies and restrictions have a direct effect on human use of the environment. In this study, national parks and the WSC 1 are defined as restricted areas under the market-based and conservation scenarios, where additional agricultural development and settlement are prohibited. LU/LC specific conversion rules indicate the annual dynamics of the land-use predictions. The relative elasticity values range from 0 (easy conversion) to 1 (irreversible change). LU/LC conversions were defined in a conversion matrix and cost for investment was included. For instance, human settlement is not likely converted to other classes due to permanent features, but cash crops are easily converted to maize or rubber plantations based on market demand. The local knowledge gathered during the consultation workshop revealed that abandoned areas of shifting cultivation take at least 5 years before regenerating to close crown-cover condition, while newly planted forest and miscellaneous land use will take at least 7 and 10 years. Based on visual image classification by the Royal Forest Department [9], closed crown-cover is categorized as forest although its quality is considerably far from the natural condition [49].

This study also assessed landscape structure and fragmentation using FRAGSTATS (version 4) [50]. The following indices were calculated (1) total area, (2) number of patches, (3) mean patch size, (4) largest patch index, (5) mean core area (area within a fragment located beyond a specified edge distance, 1 km for this study), (6) total core area, and (7) mean nearest neighbor distance index to measure patch isolation.

### 3.3. Calculation of Mean Species Abundance

The InVEST Global Biodiversity (GLOBIO) model [51] was selected to assess biodiversity response to incremental changes in land use. The original version, Global Biodiversity Model framework or GLOBIO3, was developed by the United Nations Environmental Program [52] to determine cause-and-effect relationships between five human-induced land-use changes and biodiversity, measured by mean species abundance (MSA). MSA is an aggregate estimate of species abundance relative to the pristine reference condition. The InVEST GLOBIO is appropriate for Nan province because it can represent biodiversity across all taxa, while monitoring data are typically available for charismatic megafauna (e.g., tiger, elephant, hornbill, and gibbon) in protected areas [53].

We assessed land-use change, including fragmentation and infrastructure development, and their impacts on biodiversity in the past (1977), recent present (2016) and future (2030), using the following equation developed by [52]:

(3) $${\text{MSA}}_{\text{xi}}={\text{MSA}}_{\text{LU}}\text{i}\times {\text{MSA}}_{\text{Fi}}\times {\text{MSA}}_{\text{Ii}}$$

where MSA_xi_ is the mean species abundance at a pixel as a function of land use (LU), fragmentation (F), and infrastructural development (I). MSA values range from 0 (completely disturbed) to 1 (intact) relative to the pristine stage of a particular LU/LC class. Climate change and nitrogen (N) deposition [52] were excluded in the InVEST GLOBIO model because these two pressures are suitable for global scale [54], Similar to the Dyna-CLUE model, all spatial analyses were calculated at a resolution of 200 × 200 m.

#### 3.3.1. MSA Impact from Land Use

MSA_LU_ values that relate to intensity of management or human uses were obtained from literature reviews [52]. To ensure consistency, we reclassified the original LU/LC maps [40–42]. See Supplement 1. In addition, we also conducted field surveys to measure plant diversity to assess the percentage of similarity between different degrees of human use of the LU/LC classes and intact ecosystems (i.e., evergreen forest and deciduous forest) using the Sørensen coefficient [55]. The derived average values used for paddy fields, maize area, cash crops, perennial trees, evergreen forest, deciduous forest, forest plantation, settlement and infrastructure, miscellaneous land uses, water body, and shifting cultivation were 0.1, 0.2, 0.2, 0.4, 1.0, 1.0, 0.3, 0.05, 0.2, 0.0 (not evaluated), 0.2, respectively. MSA_LU_ value for water was assigned as 0.0 because we calculated MSA values for terrestrial ecosystems only [51,52].

MSA_LU_ values incorporate the influence of human management. To distinguish between primary vegetation (more pristine), grazed grasslands, and synthetic pastures (deforested areas used for pasture), we compared the potential vegetation map (SYNMAP) generated by Jung et al. [56] to the 2016 LU/LC classes rather than Ramankutty and Foley [57] who interpreted classes from moderate resolution imaging spectroradiometer (MODIS) data. SYNMAP is a new 1–km resolution global land-cover product with improved characteristics for land-cover model parameterization. The overall advantage of SYNMAP is that all classes are properly defined in terms of plant communities.

In Nan Province, local people usually raise their cattle (domestic cows and water buffalo) in the forest (open-grazing system). In addition, they keep cattle away from cultivation areas to avoid crop damage [36]. The areas the cattle graze usually consist of patches of herbage and patches of grass found in forest gaps or deciduous forests (personal communication with local stakeholders). Therefore, we used the Normalized Differential Vegetation Index (NDVI) [58] to define pasture coverage because the NDVI is a good predictor of forage abundance [59] where ground-based methods are not practical over extensive geographic areas.

We extracted a cloud-free sub-scene of Landsat-8 TM (path/row 129/046, 129/047, 130/046, and 130/047 taken in February, March, and April 2018). The calculated NDVI values ranged between −0.44 to 0.83. Water has an NDVI value less than 0, bare soils between 0 and 0.1, deciduous forests/shrubs between 0.2 and 0.5, and dense vegetation greater than 0.5. We normalized the derived NDVI values between 0 and 1 (see equation below).

(4) $$\text{Normalized}\;\text{NDVI}=\frac{\left(ndvi_{i}-ndvi_{\text{min}}\right)}{\left(ndvi_{\text{max}}-ndvi_{\text{min}}\right)}$$

where ndvi_i_: input ndvi value ndvi_min_: minimum value of derived ndvi values (−0.44) ndvi_max_: maximum value of derived ndvi values (0.83)

The normalized NDVI subtracted from 1 was masked by the LU/LC grid for natural forest and plantation to determine the pasture area for InVEST GLOBIO. A pixel is defined as “livestock grazing” if the cell is greater than 0.2 (equivalent shrubs, deciduous forest, and open woodland). If the grassland pixel is lower than the grazing threshold, it will be defined as non-pasture use or dense vegetation, where young biomass usually occurs, in top canopy.

Cropland intensification is only calculated in the MSA_LU_ and does not affect the configuration of natural forest (MSA_I_) and the fragmentation calculated for MSA_F_. The proportion of agriculture intensity values rank between 0 and 1. Higher values are associated with intensive management (e.g., water and fertilizer supply and plowing). We defined paddy fields as “intensive agriculture” because they are irrigated and frequently fertilized [60].

#### 3.3.2. MSA Impact from Fragmentation

The InVEST GLOBIO model uses a fragmented forest quality index (FFQI) [60] to analyze forest fragmentation and assign different use categories based on FFQI, with primary forest above a user-defined threshold. This approach assumes that pristine forests are more likely to be found in large, contiguous forest patches with high species richness as defined in the species-area relationship [61]. The FFQI estimates the relative effect of fragmentation with a Gaussian smoothing function (Supplement 2). Although the method is different from the original GLOBIO framework, the results of FFQI are an accurate approximation of the more cumbersome patch-based approach [62].

#### 3.3.3. MSA Impact from Infrastructure

The impact of MSA_I_ is determined by ecosystem distance from anthropogenic classes. All LU/LC classes related to infrastructure (including settlement, water body and miscellaneous) were aggregated into a “man-made” class, while the vegetation classes, were subdivided into three types: tropical forest, temperate or boreal forest, and grassland or cropland [51,52].

The width of a zone of impact depended on the land-use type and distance to roads, [52]. See Supplement 3. We only considered the paved roads obtained from Highway Department because unpaved roads were not mapped. Human settlement was ignored because it is associated with the paved roads and therefore was a known covariate.

### 3.4. Assessing Biodiversity Hotspots

Biodiversity hotspots are defined as areas with substantial number of species, especially threatened and endemic species, which are vulnerable to anthropogenic threats such as deforestation, hunting, and farming [63,64]. This concept has been adopted and used to set priorities for biodiversity conservation and establishment of protected areas at local and regional scales across the globe [65,66]. Therefore, we used deforestation vulnerability and the MSA to identify biodiversity hotspots in Nan province. Non-forest LU/LC classes in 2030 derived from the three scenarios were overlaid with 2016 forest LU/LC classes (evergreen and deciduous forest classes) to identify the levels of deforestation vulnerability. Pixels predicted to be loss forest area in 2030 under 3 scenarios were assigned as 3 (highly vulnerable), 2 scenarios for 2, 1 scenario for value of 1, and none for 0. Meanwhile, the MSA values at the baseline (2016) were also reclassed into four classes: low as value of 0 (MSA: 0.0–0.2), medium as value of 1 (MSA: 0.2–0.4), moderately high as value of 2 (MSA: 0.4–0.6), high as value of 3 (MSA: >0.6). The deforestation vulnerability and MSA class maps were combined and categorized into 3 classes of biodiversity hotspots: low (values 0–2), medium (values 3–4), and high (values 5–6).

## 4. Results

### 4.1. Predicted Land-Use and Landscape Changes

The list of independent variables and their regression coefficients for each land-use class are shown in Table 2. Not all independent variables had a significant effect on the determination of land-use type, and the effect of each variable was not consistent across land-use types. Sandy loam soil was only significant for perennial trees, while population density seems significant for many land-use types. Furthermore, silt clay soil was significantly (positively) correlated with paddy, maize, perennial trees cultivation and deciduous forest. However, clay loam texture was negatively correlated with paddy but was a positive factor for maize and perennial cultivation. Among 17 independent variables, slope, mean temperature and distance from paved road contributed to all land-use classes. Higher altitude, steeper slope, and increased distance from streams, as well as inaccessibility were positively correlated with stabilizing the evergreen forest. In contrast, areas that are situated in clay and clay loam soils, accessible from roads, and at low altitude are suitable for paddy, maize, and perennial trees (Table 2). Soil characteristics are not significant factors for water body, settlement, and miscellaneous land uses.

Using the receiver operating characteristic area under curve (AUC) to evaluate the goodness-of-fit of a logistic regression model [67], the predicted models were outstanding for paddy (AUC > 0.9), excellent for perennial trees, forest plantation, human settlement, miscellaneous land use and water body (0.8 ≤ AUC < 0.9), and very good for the remaining land-use classes (0.7 ≤ AUC < 0.8), except cash crops (good). Relatively poorer performance for cash crops may be due to their lack of clustering on the landscape, as they occur in all soils and altitudes.

**Figure 3.**
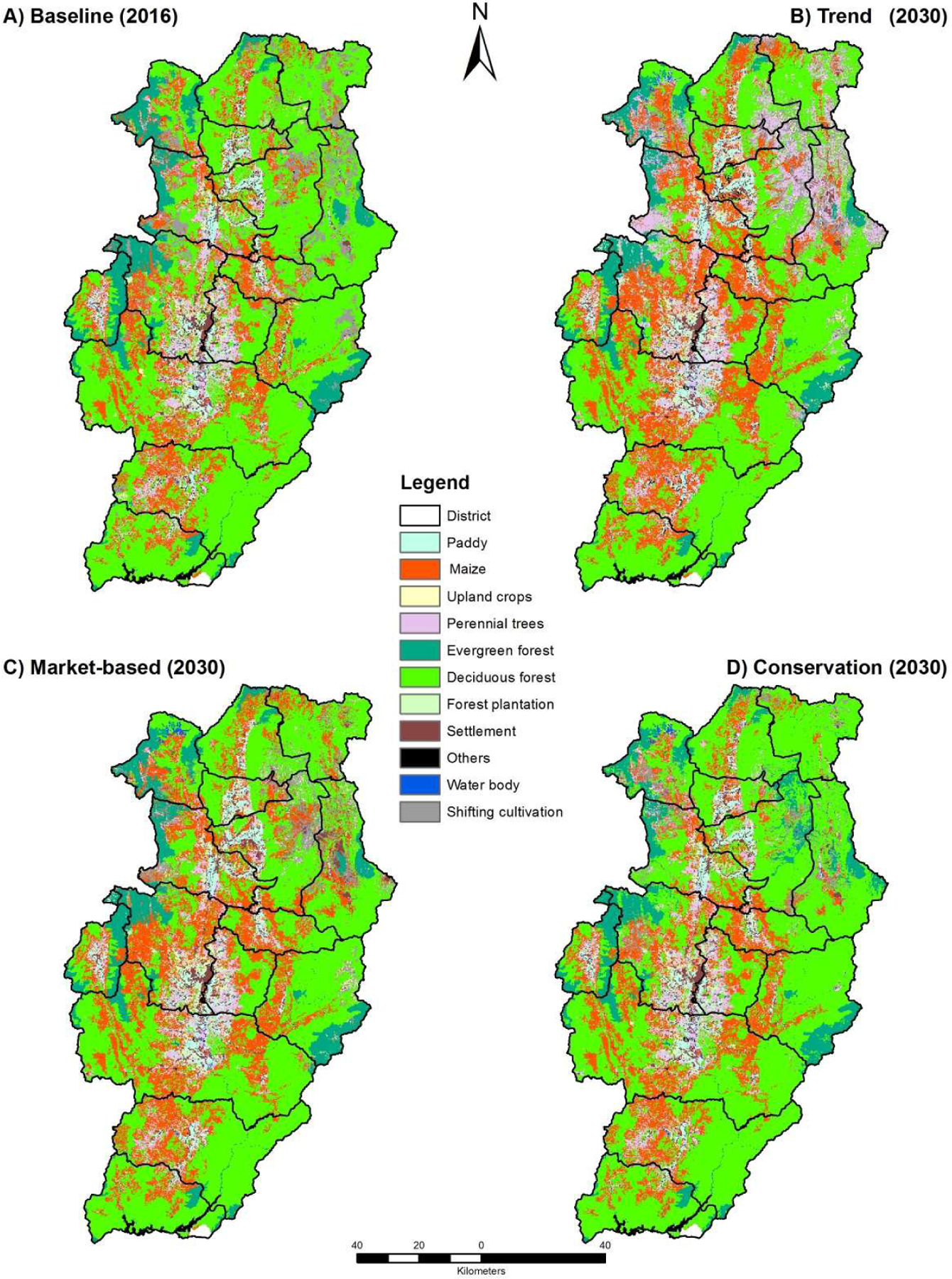
Predicted LU/LC in 2030 under different land-use scenarios.

The simulated LU/LC maps for 2030 for the three scenarios are shown in Figure 3. The trend scenario predicts expansion of rubber plantations that will lead to more deforestation in the northern part of the province (i.e., Chalerm Phrakiat, Pua, Song Kwae and Tha Wang Pha districts) but only a small increase was predicted in the south (Figure 3B). Maize cultivation was also predicted to increase from the existing cultivated areas into new areas province-wide.

The market-oriented scenario predicted moderate land conversion for maize and cash crops. The expansion of these annual crops is distributed in the east and the west similar to the trend scenario, but does not include protected areas (restriction policy) (Figure 3C). Large areas of shifting cultivation situated in high altitudes were predicted to return to forest areas either via natural regeneration (5-year duration) or reforestation practices. Paddy fields are predicted to expand in the valley along both sides of the Nan River to reduce dependence on imported rice.

Future LU/LC for the conservation scenario was similar to the baseline (2016). This scenario assumed less demand for agriculture and rubber plantations, public awareness of and the need for environmental conservation, as well as low rubber prices. Most of the shifting cultivation areas situated in north converted to forest cover (Figure 3D), including Chalerm Phrakiat, Pua, Bo Klue districts. The amount of paddy fields, maize and rubber plantations was similar to the baseline.

### 4.2. Altered Landscape Pattern

FRAGSTATS analysis revealed that the number of forest patches increased more than two-fold over the last 7 years, and mean patch size decreased about 62% compared to year 2009. Over the next 14 years of simulation (2016–2030), the number of patches would increase from 2075 at baseline to 2319 for the trend scenario. There will be a similar number (2127 patches) for the conservation scenario (Table 3). The market-based scenario has least number of patches (1,911) due to small remnant forests outside protected areas that would be converted for agriculture use, while isolated forest patches inside protected areas would be connected through natural regeneration and plantation (Table 3). The number of patches generally corresponds with mean patch size index, which changed from 363 ha in 2016 to 259 ha, 364 ha, and 385 ha for the trend, market-based, and conservation scenarios, respectively. Although the mean patch size for the market-based is nearly identical to the baseline, largest patch index and the core area of a forest patch declined substantially from baseline for the trend scenario and declined to a lesser extent for the market-based scenario, while substantially increasing for the conservation scenario. The conversion of small forest remnants nearby existing cultivation areas to meet agricultural demands under the trend and market-based scenarios explains these patterns. In contrast, fragmented forest patches become connected through natural regeneration or forest plantation under the conservation scenario.

The agricultural landscape would also shift to higher elevation. Existing rubber plantations (perennial trees) were found at mean elevation 253 m above sea level in 2009, 620 m in 2016, and would further shift to higher elevation (943 m) under the trend scenario, with similar results for the market-based and conservation scenarios. A substantial increase in rubber plantations would be expected in WSC1 and WSC2, which are headwater watersheds [61], under the trend scenario (Table 4). Although the conservation scenario would result in shifting the mean elevation for cash crops over 400 m similar to the trend scenario, cash crops occupied half of the baseline amount and approximately 22% of the market-based scenario. The extent of maize cultivation would slightly decrease under the conservation scenario (Table 4), but it would increase substantially under the trend and market-based scenarios, while remaining at the same elevation or moving to lower elevations due to restriction policies. Human settlement would expand to higher elevation due to the assumed expansion of infrastructure under all scenarios. Shifting cultivation substantially increased in WSC1 and WCS2 during 2009–2016, but forest will regenerate under the conservation scenario.

### 4.3. MSA Values

The overall MSA for Nan Province in 2009 was 0.41 and declined to 0.36 in 2016 or reduction of 12% (Table 3). MSA will decrease to 0.29 in 2030 under the trend scenario. The market-based scenario predicted a similar MSA value to baseline, while the conservation scenario predicted an increase of 11% from the baseline, returning to the MSA in 2009. Decreased MSA was mainly caused by forest fragmentation across all periods and scenarios, especially under the trend scenario. These findings agreed with the FRAGSTATS analysis, which indicated that contiguous forest blocks would become smaller and fragmented. The impact of infrastructure development on MSA was dominant for the baseline and all scenarios. The length of paved road increased 37% from 1977 to 2016 [36], substantial expansion of maize and rubber plantations contributed to future MSA loss of 26% (conservation) and 41% (trend).

**Figure 4.**
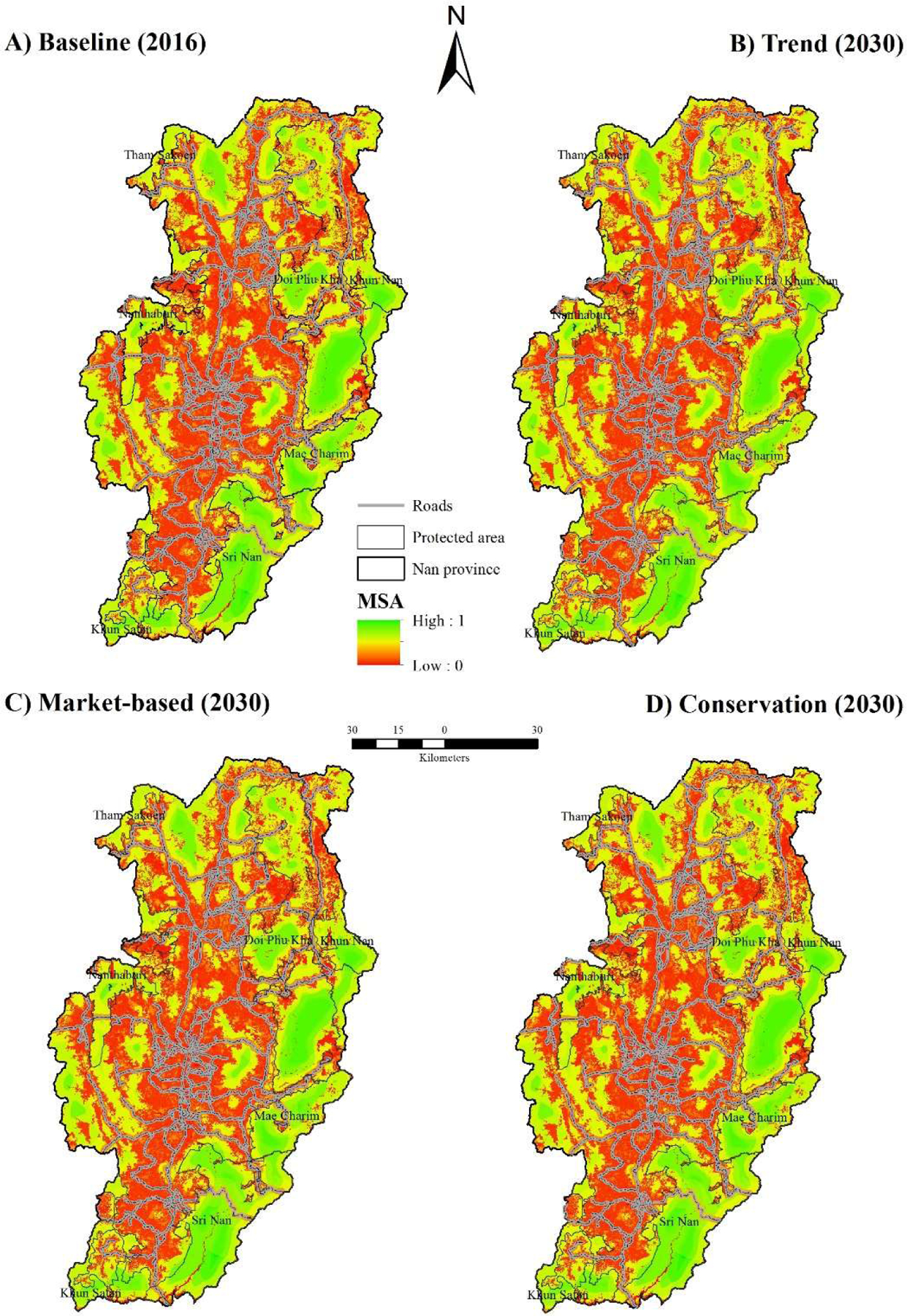
Predicted MSA values under three land-use scenarios (2030).

Greater MSA values are generally distributed in the west and in the east of Nan Province with higher elevation and inaccessible areas (Figure 4). Steep terrain deters human settlement and agriculture. The highest MSA value of 0.52 was predicted at Na Mun District, followed by Mae Charim (0.49) and Bo Kuai (0.45). Lower MSA values (0.21–0.26) were predicted at Muang Nan, Tha Wangpha, Chiang Klang and Phu Phiang Districts. These Districts are in lower elevation and have greater population density. The trend scenario predicted considerable decrease in MSA at Muang Nan, Pua, Tha Wangpha, Bo Kuai and Phu Phiang Districts largely due to deforestation. For example, MSA in Pua and Bo Kuai would decrease by half from the baseline due to deforestation alone.

Although predicted overall MSA (0.35) under the Market-based scenario was comparable to baseline (0.36), LU/LC patterns differ across the province for each. Muang Nan, Ban Luang, Tha Wangpha, Thung Chang, Bo Kuai and Phu Phiang Districts would lose biodiversity, while Mae Charim and Charoem Phrakiat Districts would gain biodiversity due to the contribution of protected areas.

### 4.4. Contribution of Protected Areas

Doi Phu Kha national Park was the first protected area in Nan Province, established in 1999. Currently there are five national parks covering approximately 31% of the province’s area and two national parks in preparation (Table 5), with larger areas of greater MSA in protected areas (Figure 4). In addition, average MSA in protected areas in 2016 was 0.57, which was 0.21 more than MSA for the Province. Decreased MSA predicted under the trend scenario is an outcome of deforestation and the assumption of lack of enforcement of logging restrictions in protected areas.

### 4.5. Biodiversity Hotspots

**Figure 5.**
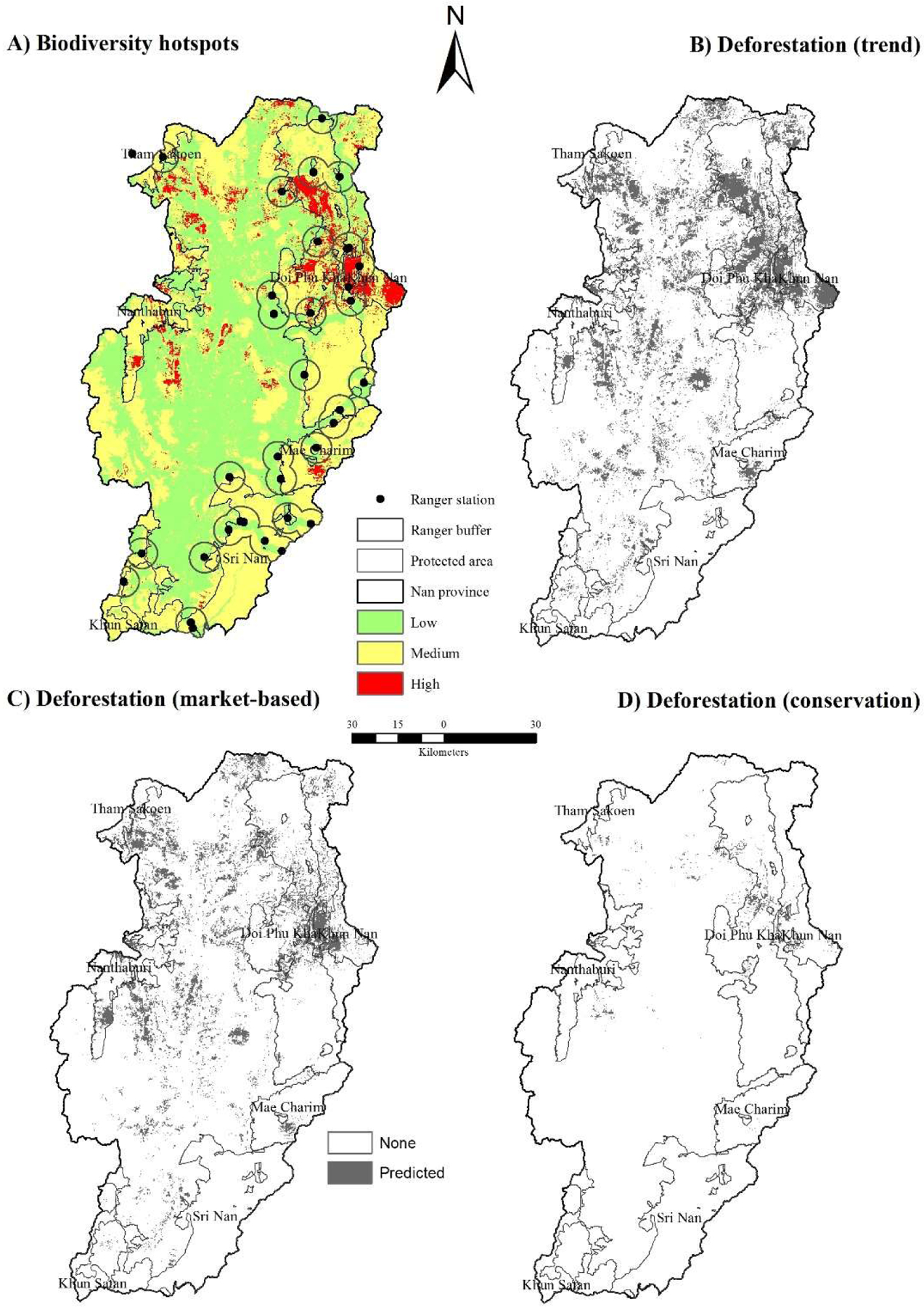
Biodiversity hotspots and deforestation in 2030 under different scenarios. Note: Locations with buffers are patrolling radius distances of 5 km from each ranger station.

Based on deforestation vulnerability and MSA (in 2016) categories of low, medium, and high, biodiversity hotspots cover 5,530, 5,911, and 720 km^2^ or 46%, 49% and 6% of Nan Province. Protected areas account 721 km^2^ (low), 3,126 km^2^ (medium), and 10,171 km^2^, (high) biodiversity hotspots (Figure 5). Approximately 56% of high biodiversity hotspots were predicted inside Doi Phu Kha, Khun Nan and Mae Charim National Parks in the west of Nan Province, with small patches scattered to the south of Tham Sakorn and Nanthaburi National Parks. About three quarters of Nan Province were classified as moderate biodiversity hotspots, with 53% located inside 7 national parks. Low biodiversity hotspots, the combination of low risk of deforestation and low biodiversity, accounted for 10% of the province’s area mainly located in the lowlands along both sides of Nan River. These areas are densely populated and have been converted to food cultivation.

## 5. Discussion

### 5.1. Dissimilarity of Land-Use Drivers at National and Provincial Levels

Agricultural and economic development in Thailand has gone through three stages, from subsistence agriculture to manufacturing and service. Thailand is currently in the fourth stage in which the economy moves towards value-based and innovative products [8]. Economic growth has helped lift millions of people out of poverty into the middle class; however, this progress had negative consequences on biodiversity. Thailand safeguarded its terrestrial environment through economic transformation with the 40% forest cover target [8], and forest cover has remained at 31–33% nationwide since 2000 [11].

However, economic development and forest quality are not the same across the country. At national level, the agricultural sector declined dramatically over the last four decades and now accounts for less than 10% of the GDP [6]. In contrast, agriculture doubled in Nan Province, from 16% of the GPP in 2000 to 32% in 2016, and this trend is likely to continue due to private business interests in maize cultivation and provincial policies [36]. Approximately half of the agricultural population lives in the highlands because it is there only source of income and support [68–70]. This rural, mainly tribal population lacks other economic opportunities and has less access to education [7].

The Nan Development Plan aims to increase commodity production not only for those with economic means but also to improve the livelihood of rural people. Reduction of the poverty ratio by 4.4% in Nan Province during 2015–2016 was largely due to increased income from the agricultural sector [36]. Once a stable and profitable source of income, rubber prices fluctuated during this decade from a high of USD 4.40 per kg in 2011 to less than USD 1.50 per kg since 2014 [34]. In addition, the current government target is to reduce rubber plantations areas by 30% in northern Thailand to decrease supply. Maize cultivation has increased due to attractive market prices and support from large agriculture companies (i.e., loans, seeds, fertilizer, transportation) through contract farming schemes [30]. Biodiversity has suffered though, because most of this newly cultivated land is in forest reserves and partially in protected areas. Although the government (Cabinet Resolution on 30 June 2000) allows farmers there to settle and cultivate without land tenure, farmers opt for the perceived security of cash crops, especially maize [71].

It should be noted that the three future land-use scenarios in 2030 defined by the stakeholders from relevant local and national levels may change due to uncertainty and dynamics of socio-economic drivers and provincial and national policies. Thus, it is not able to perform a sensitivity analysis due to limited scenario definitions. However, the evaluation results of the logistic regression models using the AUC [30,67] indicated that the performance of predicted probabilities of land-use classes were excellent and very good, except cash crops (good). In addition, the results of this research indicated that the implementation of the 40% forest cover target [8], need to take into account the variations of land characteristics and socio-economic drivers at local level.

### 5.2. Biodiversity Conservation Implication

Agriculture expansion not only reduces forest area but also fragments forest cover [13,14] and diminishes biodiversity [14,17,47]. FRAGSTATS analysis of past, present and future land-use scenarios revealed that the number of forest patches increased more than two-fold as of 2009 and would continue to increase under the trend scenario (Table 3). Average mean patch size, largest patch index and total and mean core area size would substantially decrease leading to further fragmentation.

MSA impact from forest fragmentation ranked as the largest contribution to biodiversity loss in the past, present and future, followed by land use change and infrastructure for the entire Nan Province (Table 3). However, this was not the case for protected areas. Roads through protected areas are in intact forests and provide accessibility for hunting of mammal and bird populations [20]. Thus, MSA impact from infrastructure (roads) within protected areas is ranked the largest contribution to biodiversity loss among the three pressures (Table 5).

Establishment of protected areas and their effective management are key to protecting the remaining forest cover and to maintaining biodiversity. Forests are 89% of protected areas in 2016 and would increase to 91% under the conservation scenario (two more national parks and restriction policies). However, decreased forest cover and MSA are predicted under the trend scenario because of poor enforcement of restriction policies on forest encroachment, wildlife poaching and subsistence or overexploitation. Furthermore, ineffective management would accelerate pressure on biodiversity for human needs such as fuelwood collection and grazing [72].

Currently, 35 ranger stations, check-points and headquarters have been established to safeguard biodiversity in five national parks (excluding Nanthaburi and Sri Nan National Parks). With a patrolling radius distance of 5 km from each ranger station [12,18], priority areas require additional protection measures to be effective such as patrolling, community-outreach program, and expansion of protected areas (conservation scenario) (Figure 5). Otherwise, protected areas remain at risk of agricultural encroachment. Proactive community education and outreach activities around protected areas, ecotourism and non-timber forest products can suppress poaching and initiate wildlife recovery in Thailand [73].

Beside restriction policies, economic incentives to reduce highland maize farming and encourage reforestation in degraded headwatersheds should be investigated. These include improving crop productivity and employing farmers to work in reforestation and park activities [71]. In addition, the government may compensate farmers to change from maize plantation on steep slopes to multi-layer cropping to enhance watershed services.

Agricultural land tended to shift to higher altitude in headwatershed areas, under the trend and market-based scenarios (Table 4), due to poor restriction policies and high demand for agriculture land. Vulnerable deforestation areas were still intact and contained high biodiversity (Figure 4). Further deforestation not only increases biodiversity loss but also degrades watershed services such as the provisioning of clean water and sediment retention [22,23].

### 5.3. Additional Research Needs

We used the InVEST GLOBIO model [50] to quantify the relative contribution of land-use change, fragmentation, and infrastructure on MSA. MSA impacts from N deposition and climate change proposed in the original GLOBIO3 model [52] were excluded. In addition, we used remote sensing techniques to determine potential pasture lands and conducted field surveys to obtain local MSA impacts rather than using the default data.

Additional pressures should be investigated in future research, including hunting, non-timber product collection, unpaved roads, and projected population. The abundance of bird and mammal populations in tropical ecosystems has a strong relationship with hunting pressure [20]. Although non-timber forest products (e.g., bamboo, edible plants, honey) play a role in rural livelihoods and can contribute to sustainable forest management, there are also negative impacts on biodiversity [73]. Issues include overharvesting, opportunistic wildlife poaching, and illegal logging. Unpaved roads should be mapped because they also provide accessibility to encroachment and hunting [20]. The population in Nan Province is predicted to decrease after 2020 in particular in the cities more than mountain areas [7] where deforestation is expected (Figure 3).

## 6. Conclusions

This study demonstrated that land-use policy and practice at national and provincial levels have contrasting outcomes. Nationally, forested, and agricultural LU/LC have stabilized because of movement away from agriculture-based economy. Nan Province is being left behind though in the national economic transformation. National LU/LC policy contrasts with provincial realities that include less suitable arable land, greater slopes, and farmers with fewer economic means. These factors will only increase pressure on forest biodiversity. Agriculture is expected to increase because it is the main source of household food and income and other economic opportunities do not yet exist. The market-based scenario was guided by provincial policies that aimed to balance economic and social development and biodiversity conservation. The model predicted similar biodiversity to baseline (2016), while the GPP from agricultural sector increased 2% from the baseline. The conservation scenario aimed to maintain existing forest cover, add more protected areas, and rehabilitate degraded areas in headwater watersheds. This ambitious conservation target appears less sustainable than the market-based scenario and will conflict with economic and social demands. Nevertheless, the three scenarios in Nan Province (trend, market-based and conservation) derived from socio-economic-environmental factors target more forest cover than the national 40% forest cover policy. Thus, national policy should be more flexible and take into account local contexts.

Currently, the government and public are concerned about the consequences of deforestation on biodiversity and ecosystem services. The Thailand 20-year National Strategic plan (2017–2036) was approved by the government to guide six strategic areas: security, competitiveness enhancement, human resource development, social equality, green growth and rebalancing and public sector development. Nan Province has been selected as a pilot project under the green growth (natural resources and environment component). Numerous projects (e.g., multi-layer cropping, payment for ecosystem services, conservation awareness, forest patrolling) are being implemented. The results of this study effectively support these initiatives for long-term biodiversity conservation and sustainable land-use management in Nan Province.

This study indicated that integrated land-use change and biodiversity models was very useful not only to predict future land use based on various socio-economic drivers and demands, but also to visualize vulnerable deforestation areas and to priority areas for biodiversity conservation on the landscape. Therefore, policy makers and practitioners can foresee the predicted issues and can allocate limited resources efficiently for long-term conservation planning. In addition, both models require moderate data inputs and the approaches used in this research can be applied in other regions to support biodiversity conservation.

## Supplementary Material

Sup1

## Figures and Tables

**Table 1.** Extent of land-use/land-cover classes in 1977, 2009 and 2016.

LU/LC	1977		2009		2016		Change 2009–2016		
	(ha)	%	(ha)	%	(ha)	%	Ha	%	Annual Rate
Paddy^1^	52,540	4.32	37,604	3.1	38,860	3.2	1256	3.34	0.47
Maize	5590	0.46	136,708	11.2	212,720	17.5	76,012	55.60	6.52
Cash crops^2^	2310	0.19	2116	0.2	5352	0.4	3236	152.93	14.18
Perennial trees^3^	4014	0.34	35,256	2.9	75,008	6.2	39,752	112.75	11.39
Evergreen forest	108,037	8.91	105,892	8.7	97,436	8.0	−8,456	−7.985	−1.18
Deciduous forest	1,020,080	83.87	744,316	61.2	656,072	53.9	−88,244	−11.86	−1.79
Forest plantation^4^	240	0.02	25,164	2.1	19,388	1.6	−5,776	−22.95	−3.66
Settlement	11,310	0.93	20,520	1.7	21,896	1.8	1376	6.71	0.93
Others^5^	n/a	-	6876	0.6	3876	0.3	−3000	−43.63	−7.86
Water^6^	4140	0.34	7440	0.6	8316	0.7	876	11.77	1.60
Shifting cultivation^7^	7420	0.61	94,368	7.8	77,336	6.4	−17,032	−18.05	−2.80
Total	1,216,260	100.00	1,216,260	100.0	1,216,260	100.0	0		

Source: [40,43,44]. Notes:

1 irrigated rice,

2 all cash crops, excluding maize,

3 include fruit trees and rubber plantations,

4 mono forest tree plantation (e.g., teak, eucalyptus),

5 bare soil and miscellaneous,

6 streams, rivers, lakes/dams,

7 slash and burn forest and swidden cultivation.

**Table 2.** Beta values of significant variables for regression models related to each land-use type.

Variables	Paddy	Maize	Cash Crops	Perennial Trees	Evergreen Forest	Deciduous Forest	Forest Plantation	Settlement	Misc.	Water	Shifting
Clay soil	ns	2.084	ns	1.169	ns	1.554	ns	−0.506	ns	ns	ns
Clay loam soil	−0.985	2.729	ns	0.379	ns	2.144	ns	−1.026	ns	ns	ns
Loam soil	ns	2.293	ns	ns	ns	1.181	ns	ns	ns	ns	ns
Slope complex	−1.218	2.939	ns	ns	ns	2.656	ns	−1.700	ns	ns	3.881
Sandy clay loam soil	ns	1.953	ns	0.740	ns	1.323	ns	ns	ns	ns	ns
Sandy loam soil	ns		ns	2.483	ns	ns	ns	ns	ns	ns	ns
Silt loam soil	ns	1.030	ns	1.353	ns	ns	ns	ns	ns	ns	ns
Silt clay soil	1.262	0.810	ns	ns	ns	0.63	ns	0.743	ns	ns	ns
Silty clay loam soil	0.992	ns	ns	ns	ns	ns	ns	ns	ns	ns	ns
Altitude (m)	−0.019	−0.003	ns	−0.004	0.004	0.004	ns	−0.005	−0.008	−0.017	ns
Distance to road (m)	−1.3 × 10^−4^	−6.6 × 10^−5^	−7.2 × 10^−5^	−9.0 × 10^−5^	7.5 × 10^−5^	5.3 × 10^−4^	−1.5 × 10^−4^	−1.5 × 10^−4^	−1.5 × 10^−4^	5.6 × 10^−5^	−1.2 × 10^−4^
Distance to stream (m)	ns	ns	−7.9 × 10^−5^	ns	2.7 × 10^−5^	−1.8 × 10^−5^	−7.1 × 10^−5^	5.8 × 10^−4^	−5.7 × 10^−5^		7.4 × 10^−5^
Slope (%)	−0.351	−0.067	0.079	−0.088	0.027	0.069	−0.125	−0.216	−0.197	−0.169	−0.008
Mean temp. (°C)	−0.295	−0.020	−0.029	−0.025	0.037	0.005	0.061	−0.075	−0.111	−0.203	0.027
Annual rainfall (mm)	−006	−0.004	−0.005	ns	0.006	−0.002	ns	ns	ns	ns	0.007
Agr. pop. density (hh/km^2^)	−0.013	0.006	−0.037	0.034	0.037	−0.030	0.053	ns	−0.016	−0.049	0.038
Pop. density (ind./km^2^)	ns	−0.001	ns	−0.001	−0.016	−2.2 × 10^−5^	ns	0.001	0.001	ns	−0.008
Constant	90.645	8.91	14.861	11.237	−19.789	−2.157	−14.509	22.864	32.763	58.191	−3.534
AUC	0.942	0.717	0.693	0.834	0.788	0.723	0.879	0.898	0.876	0.897	0.759

Note: AUC = area under curve; ns = not statistically significant.

**Table 3.** Landscape indices of remaining forest area and relative contribution of different threats to the reduced MSA in Nan province during 2016–2030.

Patch Indices/MSA Values	2009	Baseline (2016)	Trend	Market-Based	Conservation
% forest area	70.00	61.95	47.36	57.15	67.02
No. of patches	853	2075	2,319	1911	2127
Mean patch size (ha)	966	363	259	364	385
Largest patch index (%)	67	55	32	44	60
Total core area (1000 × ha)	279	235	158	204	261
Mean core area (ha)	327	113	68	107	123
Mean proximity index (m)	480	475	497	489	467
Overall MSA	0.41	0.36	0.29	0.35	0.40
Remaining MSA caused by land use (MSA_Lu_)	0.77	0.69	0.59	0.66	0.74
Remaining MSA caused by infrastructure (MSA_I_)	0.75	0.84	0.85	0.84	0.84
Remaining MSA caused by fragmentation (MSA_F_)	0.60	0.54	0.48	0.53	0.58

**Table 4.** Land use in watershed classes 1 and 2 in Nan Province and mean elevation for each LU/LC.

LU/LC	2009		2016		Trend		Market-based		Conservation	
	Area (%)	Mean Elev (m)	Area (%)	Mean Elev (m)	Area (%)	Mean Elev (m)	Area (%)	Mean Elev (m)	Area (%)	Mean Elev (m)
Paddy	0.1	445	0.1	456	0.2	476	0.2	457	0.1	472
Maize	12.2	932	13.5	834	17.7	657	18.7	735	12.3	915
Cash crops	0.8	632	0.4	734	1.2	1185	0.9	832	0.2	1446
Perennial trees	0.0	253	1.9	620	9.9	943	1.9	626	2.1	654
Evergreen forest	11.2	1029	10.7	1026	9.3	1063	10.3	1149	11.5	1019
Deciduous forest	71.2	442	64.5	1041	53.4	972	61.2	992	69.4	1032
Forest plantation	0.2	589	0.3	541	0.4	555	0.3	502	0.3	704
Settlement	0.2	477	0.2	606	0.6	793	1.0	706	0.4	836
Others	0.1	390	0.0	400	0.0	302	0.0	299	0.0	322
Water	0.2	843	0.2	400	0.3	409	0.3	386	0.3	407
Shifting cultivation	3.8	350	8.0	956	6.9	1088	5.2	982	3.4	1,111
Total	100.00		100.0		100.0		100.0		100.0	

Note: Total area of watershed classes1 and 2 covers 879,832 ha (approximately 72% of Nan Province).

**Table 5.** MSA within protected areas and their contribution to provincial MSA.

	2009		2016		Trend		Market-based		Conservation	
	MSA	% cont.	MSA	% cont.	MSA	% cont.	MSA	% cont.	MSA	% cont.
Protected areas	3		5		7		7		7	
Area (km^2^)	3155	26	3750	31	4430	37	4430	36	4430	36
% forest cover	85	31	89	42	77	64	87	53	91	40
Overall MSA	0.61	38	0.57	56	0.49	58	0.57	58	0.60	52
MSA_LU_	0.93	31	0.89	45	0.81	48	0.89	47	0.93	39
MSA_I_	0.77	33	0.72	46	0.64	46	0.72	47	0.75	45
MSA_F_	0.82	28	0.83	23	0.84	23	0.83	40	0.83	43

Note: MSA = Mean Species Abundance, % cont. = percentage found inside protected areas against the provincial areas.
