# Supplementary material for: Land-Use/Land-Cover Change from Socio-Economic Drivers and Their Impact on Biodiversity in Nan Province, Thailand
Source: Sustainability. Author manuscript; Available in PMC 2020 Dec 21. (PMC7751623; doi:10.3390/su11030649)
Supplement: Sup1 [file NIHMS1541432-supplement-Sup1.pdf]

Supplement 1. LDD LU/LC conversion to GLOBIO LU/LC and remaining MSA affected by land use.

LDD LU/LC class	GLOBIO LU/LC classes	MSA_{LU} (default)	MSA_{LU} (Nan)*
Paddy	Intensive agriculture	0.1	0.1
Maize	Low-input agriculture	0.3	0.2
Cash crops	Low-input agriculture	0.3	0.2
Perennial trees	Agroforestry	0.5	0.4
Evergreen forest	Primary vegetation	1.0	1.0
Deciduous forest	Primary vegetation	1.0	1.0
Forest plantation	Forest plantation	0.2	0.3
Settlement	Build-up areas	0.05	0.05
Others (miscellaneous)	Man-made pasture	0.1	0.2
Water	N/A	N/A	N/A
Shifting cultivation	Man-made pasture	0.1	0.2

Note: LDD = Land Development Department; MSA_{LU} = Remaining MSA affected by land use management; N/A = Not Applicable; * derived from similarity index (Sorensen coefficient); MSA_{LU} values range from 0 (completely disturbed) to 1 (intact) relative to the pristine stage of a particular LU/LC class

Source: [45]

Supplement 1. LDD LU/LC conversion to GLOBIO LU/LC and remaining MSA affected by land use.

LDD LU/LC class	GLOBIO LU/LC classes	MSA_{LU} (default)	MSA_{LU} (Nan)*
Paddy	Intensive agriculture	0.1	0.1
Maize	Low-input agriculture	0.3	0.2
Cash crops	Low-input agriculture	0.3	0.2
Perennial trees	Agroforestry	0.5	0.4
Evergreen forest	Primary vegetation	1.0	1.0
Deciduous forest	Primary vegetation	1.0	1.0
Forest plantation	Forest plantation	0.2	0.3
Settlement	Build-up areas	0.05	0.05
Others (miscellaneous)	Man-made pasture	0.1	0.2
Water	N/A	N/A	N/A
Shifting cultivation	Man-made pasture	0.1	0.2

Note: LDD = Land Development Department; MSA_{LU} = Remaining MSA affected by land use management; N/A = Not Applicable; * derived from similarity index (Sørensen coefficient); MSA_{LU} values range from 0 (completely disturbed) to 1 (intact) relative to the pristine stage of a particular LU/LC class

Source: [52]

Supplement 2. Fragmentation effect on MSA under varying patch sizes.

FFQI	Area (km^2)	MSA_F
< 0.43	≤ 1	0.3
0.43 – 0.58	≤10	0.6
0.58 – 0.90	≤100	0.7
0.90 – 0.98	≤1,000	0.9
0.98 – 0.99	≤10,000	0.95
0.99 – 1	>10,000	1.0

Note: FFQI = fragmented forest quality index; MSA_F = Remaining MSA affected by habitat fragmentation; MSA_F values range from 0.3 (severely fragmented) to 1 (contiguous and very large habitat patch).

Source: [52, 60]

Supplement 3. Impact of infrastructure on MSA values (MSA_I).

Impact Zone	Tropical forest distance to infrastructure (m)	Temperate forest distance to infrastructure (m)	Grassland & cropland distance to infrastructure (m)	MSA_I
High impact	<1000	<300	<500	0.4
Medium Impact	1,000-4,000	300-1,200	500-2,000	0.8
Low Impact	4,000-14,000	1,200-4,200	2,000-7,000	0.9
No Impact	>14,000	>4,200	>7,000	1.0

Note: MSA_I = Remaining MSA affected by habitat fragmentation; MSA_I values range from 0.4 (nearness to roads or infrastructure development) to 1 (no impact or remoteness).

Source: [52]

Data availability statement: The datasets generated during and/or analyzed during the research are available from the corresponding author (email: fforyyt@ku.ac.th) on reasonable request.
